# What Is Performance? A Scoping Review of Performance Outcomes as Study Endpoints in Athletics

**DOI:** 10.3390/sports7030066

**Published:** 2019-03-16

**Authors:** Benjamin P. Raysmith, Jenny Jacobsson, Michael K. Drew, Toomas Timpka

**Affiliations:** 1 Athletics Research Centre, Linköping University, 581 83 Linköping, Sweden; jenny.jacobsson@liu.se (J.J.); toomas.timpka@liu.se (T.T.); 2 Athletics Australia, Melbourne 3206, Australia; 3 Swedish Athletics Association, 120 30 Stockholm, Sweden; 4 Athlete Availability Program, Australian Institute of Sport, Bruce 2617, Australia; Mick.Drew@ausport.gov.au; 5 Australian Collaboration for Research into Injury in Sport and Its Prevention (ACRISP), Melbourne 3086, Australia; 6 University of Canberra Research Institute for Sport and Exercise (UCRISE), Canberra 2617, Australia

**Keywords:** performance evaluation, success, achievement, athletic performance, ranking

## Abstract

Purpose: This review set out to summarise, define, and provide future direction towards the use of performance outcome measures as endpoints in research performed at international benchmark events in athletics. Methods: Scoping review methodology was applied through a search of the PubMed and Sports Discus databases and a systematic article selection procedure. Articles that met the inclusion criteria underwent triage for further quantitative and qualitative analysis. A concept chart was generated to describe the methods by which performance had been measured and introduce descriptive labels for theoretical and practical application. Results: None of 2972 articles primarily identified from the database search met the triage standards for quantitative data extraction. Eleven articles were included in a qualitative analysis. The analysis identified the common methods by which performance has been measured, reported and analysed. The resulting concept chart collates labels from the qualitative analysis (categories, themes, and constructs) with sports practice labels (performance metrics, framework, and analysis). Conclusions: The state of knowledge concerning methods to employ performance metrics as endpoints in studies performed at major competitions in athletics has been summarised. Constructing a methodology that combines the performance metric variables (continuous and ordinal) that are currently utilised as endpoints remains a challenge.

## 1. Introduction

Measurements of performance success influence possibilities to participate in individual sports at the highest level through mechanisms such as qualifications to the largest competitions, sponsorship contracts, government funding of sports organisations, and subsequently funding of athletes by those organisations. Winning and personal bests are typical benchmarks of outstanding achievements in this setting. Performance success may also be regarded as the ultimate endpoint of sports medicine and epidemiological research in elite sports through identifying and investigating factors that influence performance outcome (PO). The definition of PO and its evaluation through objective methodology therefore requires consistency and consideration of the broad array of factors that contribute to that endpoint.

The methodology to evaluate or define ‘performance’ among elite individual athletes has not been settled in the scientific literature and poses a real-world challenge for sporting bodies when objectively evaluating performance. The same challenge is posed when attempting to investigate factors that influence performance. One reason for this debate is that a paradox exists whereby a subjective evaluation of an individual athlete’s PO can be dominated by the comparison with others regardless of a goal to outperform oneself [1]. Individuals competing at recreational sport level are often motivated by intrinsic factors such as psychological wellbeing, maintaining fitness, enjoyment or skill development [2,3]. Nonetheless, for individual athletes at the very highest level, performance at the key competitions is the outstanding endpoint measure for success. The international benchmark event (IBE) thus constitutes a highly suitable setting for studies of factors influencing performance among elite athletes.

Furthermore, the traditional endpoints for studies in sports medicine and epidemiology have been clinical endpoints like injury and illness [4]. However, these health factors may not be sensitive enough to capture the essence of what is required for sports performance at the very highest level. Notions such as time-loss injury have been introduced to better suit the sports context [5]. The measure of time-loss from participation may still be insufficient due to that even minor deviations from optimal health and capacity level may prove crucial to success at the highest level of sports [6]. A recent trend investigating outcomes in sports epidemiology has endeavoured to progress the traditional endpoints of injuries and illnesses towards the endpoint of ‘performance’. Injuries impair the chance of success by sportspeople [7], and time lost during a competition preparation phase due to injury and illness is associated with decreased likelihood in achieving a performance goal in athletics at IBEs [6]. The continued direction of this work to progress from injury and illness as endpoints in high performance sport requires the development of an objective methodology and working definition of ‘performance’ as an endpoint in sports medicine and epidemiology settings. This outcome would enable researchers to progress, when warranted, from injury and illness being the endpoint to investigating injury and illness as factors that may ultimately influence performance.

Athletics (track and field) is the most participated-in sport at the Olympic Games [8] and one of the highest participation sports in Europe [9]. The real-world challenge in evaluating performance objectively in athletics is demonstrated by the example where an athlete may underperform according to their meet rank yet outperform their season’s best time. This challenge is compounded in athletics by some running events having an external tactical focus towards other competitors in a race (e.g., 800 m and 1500 m) and other events an internally focused maximum effort (e.g., 100 m and javelin throw). The objective evaluation of performance at an IBE and investigation of factors that influence performance are impaired currently by the absence of a consistent working definition and methodology to do so.

Scoping reviews have recently been introduced to examine the extent, variety, and nature of research evidence on a novel topic; summarise findings from a heterogeneous body of knowledge; and identify gaps in the literature to aid the planning of future research [10]. The aim of this study was to apply scoping review methodology to examine the use of various POs as endpoints in research performed at athletics competitions of the highest level. A secondary aim is to provide directions for future work into defining PO in individual sports and the methodology to analyse performance objectively for its utilisation as an endpoint in performance evaluation, sports medicine, and epidemiology.

## 2. Methods

The Preferred Reporting Items for Systematic Reviews and Meta-analysis Protocols (PRISMA-P) guidelines [11] were followed to identify a primary set of articles for data extraction and review. The 5-step process as described by Arksey and O’Malley [12] with enhancements as described by Levac and colleagues [13] was utilised: Identify the research question, identify relevant studies, study selection, chart the data, and collate, summarise, and report the results. In the final step, the review process was supplemented by application of thematic analysis methods [14]. The PRISMA extension for scoping reviews (PRISMA-ScR) checklist was used to ensure complete and transparent reporting [10]. Before initiating the review, the protocol for the analysis was registered on the PROSPERO International prospective register for systematic reviews website (http://www.crd. york.ac.uk/PROSPERO) on 11 February 2018 (registration number: CRD42018087272).

### 2.1. Identification of Relevant Studies

The research question to be addressed by the review was: “How has performance at IBEs in athletics been objectively analysed?” The purpose of restricting the review to IBEs was to capture performances where athletes are most likely to be striving for their highest outcome of the season. The article inclusion criteria were: Published in English, reporting original research, involve athletics athletes, reporting research encompassing IBEs (International Association of Athletics Federation (IAAF) World Championships, Olympic Games, continental championships, and Commonwealth Games), and reported athletes’ performance. Articles were excluded under the following criteria: Study involved subjects under 18 years old, and the study involved a performance metric that was not ‘event outcome based’, e.g., physiological measures, laboratory tests, and physical parameter tests, e.g., jumps tests, time trials, strength tests.

A search of the PubMed, Sports Discus, and e-journals databases was conducted in February 2018 using the terms: ‘athletics’, ‘track and field’, ‘Olympics’, ‘world championships’, ‘athletics performance’, ‘achievement’, ‘programme evaluation’, ‘performance evaluation’, ‘success factors’, ‘success’, ‘achievement’, ‘scoring’, ‘ranking’ (Appendix A). All records retrieved by the search query were imported in to Endnote X7 (Thompson Reuters, Carlsbad, CA, USA) and duplicates removed.

### 2.2. Final Study Selection

The retrieved records were in the next step uploaded to Covidence software (Covidence systematic review software, Veritas Health Innovation, Melbourne, Australia. Available at www.covidence.org). Two authors (B.P.R., M.K.D.) independently reviewed titles and abstracts for potential eligibility. For the potentially eligible records, the full-text articles were thereafter retrieved and assessed according to the inclusion and exclusion criteria. The reference lists of the resulting articles were searched by the lead author (B.D.R.) for inclusion of additional articles. Any discrepancies were discussed by the reviewers (B.D.R., MK.D.). No conflicts were outstanding. The review of full-text articles revealed that those articles that reported a performance metric provided sufficient content data for a continued analysis.

### 2.3. Collating the Results

A thematic analysis was performed to assess the articles reporting on a performance metric for categories and themes using a six-stage recursive process [14]. The lead author (BR) conducted a risk of bias assessment using the Downs and Black checklist [15] for randomised controlled trails (RCTs) and the Newcastle–Ottawa scale (NOS) for assessing the quality of nonrandomised studies [16]. The Downs and Black checklist assign a score out of 31 with ≥75% as deemed to be high quality or ‘low’ risk of bias, 60–75% moderate quality, and ≤60% as low quality or ‘high’ risk of bias [17]. The NOS uses a star rating system up to a maximum score of 9 stars divided in to three classes: Selection, comparability, and outcome. The thematic analysis was thereafter performed by the lead author (BR) to summarise and coalesce the content of the articles. The information charted from the articles included the study design, country, study population, study aims, performance metric, analysis, and risk of bias assessment (on RCTs and cohort studies). Significant parts were identified and sorted into categories and themes. In the final step, a concept chart was generated through an iterative process to provide a model representation of the methods by which the assessed articles measured, reported, and analysed performance.

## 3. Results

### 3.1. Literature Search

**Figure 1:**
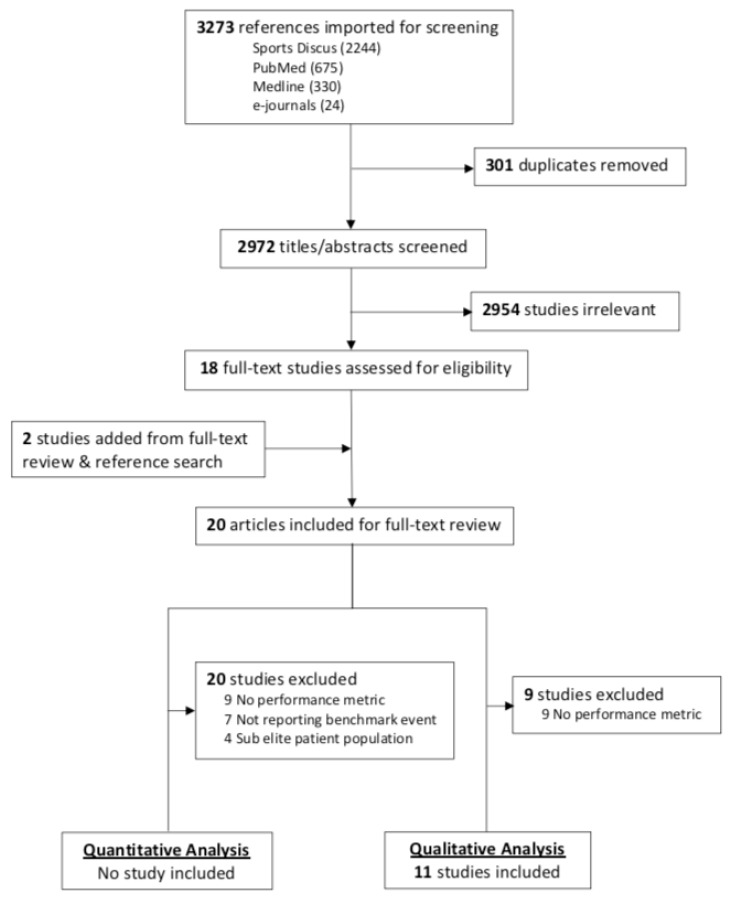
Flow chart of Preferred Reporting Items for Systematic Reviews and Meta-analysis Protocols (PRISMA) article selection process.

The initial literature search identified a total of 2972 articles for title and abstract review. Twenty articles were selected for full-text assessment with the aim to extract data for either quantitative or qualitative analysis. Nine articles did not report a performance metric, seven did not report results from a benchmark event, and four articles included a sub-elite athlete population, thus providing no articles that met the desired frame of reference for quantitative data extraction. Eleven of the 20 articles reported a performance metric that provided data for qualitative thematic analysis (Figure 1).

### 3.2. Risk of Bias, Data, and Concept Thematic Extraction

Six of the 11 articles eligible for thematic analysis (randomised controlled trial (RCT), *n* = 1; Cohort studies, *n* = 5) underwent risk of bias assessment (Appendix B). The RCT was deemed to have a ‘high’ risk of bias (55%). All cohort studies scored ≤2/4 in the ‘selection’ category relating to the studies not being designed in a fashion that included exposed and non-exposed cohorts, zero in the ‘comparability’ section for the same reason, and 3/3 in the ‘outcome’ section relating to the results recording process and subject follow-up. The remaining five articles were descriptive studies and did not undergo risk of bias assessment.

The thematic analysis of the 11 articles brought to light the reporting of performance categories, themes, and constructs to describe POs within individual sports like athletics (Table 1). Two categories were identified to define the ‘performance metrics’ that represent the event or competition result: ‘continuous’ data (*n* = 3) such as heights [18,19], times [18,19,20], distances [18,19], and conversion to IAAF points scores [18,19,20] or ‘ordinal’ data (*n* = 9) ranked against a finishing position [19,21] or performance standards including personal bests [22,23], season’s bests [23,24,25,26,27], national records [25] or world records [28]. One article reported performance metrics according to both categories, continuous and ordinal [19].

Two themes describe the ‘framework’ within which the scope of each performance metric resides: Comparison of the result or rank with other athletes (INTER-personal scope, *n* = 3) [19,21,25], or comparison of the result or rank relative to one’s own previous performance (INTRA-personal scope, *n* = 10) [18,19,20,22,23,24,25,26,27,28]. Two articles reported performance metrics within the scope of both frameworks, intra- and inter-personal [19,25].

Two constructs were identified to ‘analyse’ the themed categories: ‘deviations’ (*n* = 8) represent a divergence from a performance trend like meet, annual or career performances [18,20,23,24,27] or divergence from performance standards such as season’s best or ranking [21,25,26], and ‘associations’ (*n* = 6) between the performance metric and predetermined independent variables, such as psychological factors [19,25,28], physical or physiological factors [22], and societal factors [24,27]. Three articles analysed performance metrics by both constructs, deviations and associations [24,25,27]. Thematic extraction identified a common flow of analysing performance that established a PO for evaluation.

### 3.3. Concept Chart

**Figure 2:**
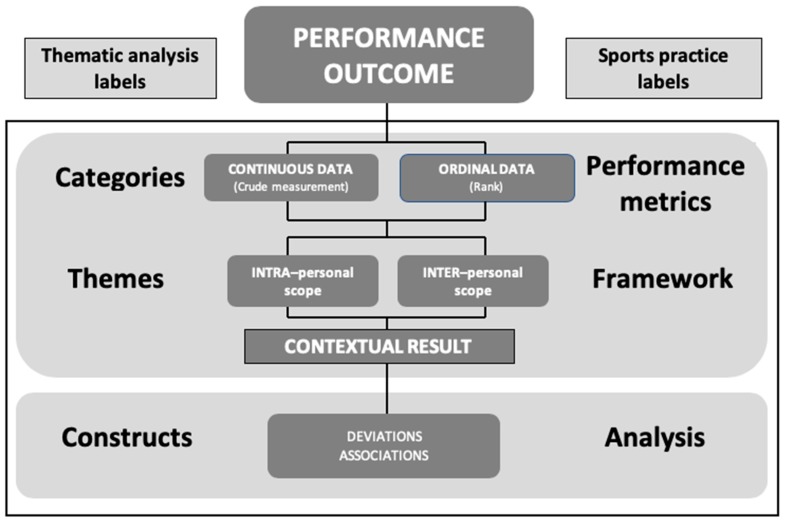
Concept chart illustrating objective methodology to identify performance outcome (PO) as an endpoint measure.

Two primary features of the concept chart define and illustrate ‘PO’ as an endpoint measure (Figure 2). ‘Thematic analysis labels’ (categories, themes, and constructs) are provided for generalisability along with ‘sports practice labels’ (performance metrics, framework, and analysis) to guide the practical application of the results from the thematic analysis.

The performance metrics ‘categories’ report the crude measurement of the event or competition result and rank. The ‘framework’ themes add scope/context to the performance metric for analysis. The resultant themed category, or ‘contextual result’, is finally ‘analysed’ using one of or several constructs. The end result of this methodology provides the ‘PO’ that may then be ‘evaluated’ by a sports federation or academic purpose according to their own objectives. This objective methodology offers consistent sports practice labels to defining PO by applying a framework to the performance metric establishing a contextual result that may then be analysed according to an applied construct.

A framework is applied to the performance metric producing the contextual result, which may then be analysed according to an applied construct.

## 4. Discussion

Despite its widespread discussion in nonacademic spheres, to our knowledge, no research has sought to use objective methodology to summarise use of POs as endpoints for studies performed at IBEs in athletics. This review reports that research to date on performance in athletics competitions at the highest level is predominated by continuous and ordinal performance metrics as endpoints for analysis within an intra-personal framework. The performance metrics have commonly been analysed by illustrating deviations from performance trends or performance standards, or by observing associations with predetermined independent variables, or both.

The framework resulting from the review also places the objectified PO as one important component within this broader appraisal process. From a sports practice perspective, performance evaluation processes have to date involved subjective evaluation according to the particular criteria that, for instance, a sports federation chooses to apply. Such evaluations require the consideration of many factors relating to an athlete’s performance and context within which that performance lies. Further, a comprehensive structured framework may never have the capacity to incorporate all of those factors.

### 4.1. The Need for an Evaluation Context

The qualitative analysis of the included articles content identified themes that provide an avenue to apply context to performance metric categories. By applying a framework to the interpretation of a performance metric, the crude measurement or finishing position can at the basic level be analysed in comparison to other athletes or within one’s own historical results. This ‘contextualised result’ provides a foundation on which further analyses may follow. Such assessments may support sports practices by focusing on deviations from a performance standard like a national or personal record, deviation from a trend like seasonal improvement, or constitute academic investigations of associations with physiological, psychological or societal factors. The framing of the performance metric with intra- or inter-personal scope thus provides a platform to analyse the contextual result through a combination of constructs that enhance the possibilities to interpret the PO as a basis for decision-making in sports practice and/or scientific inferences.

A recently proposed practical application of structured PO analysis with intra-personal scope is tracking of performances to identify unusual improvements possibly caused by doping, the so-called athlete performance module (APM) in the “athlete’s performance passport” [31]. The individual variation of performance in elite athletics athletes over a single season has been reported to be small, with a coefficient of variation ranging from 1.1 to 1.4% (90% CI: 1.0–1.6%) [32]. In the APM, performance data are modelled based on past performances, and unusual performances by an athlete trigger a more thorough testing program. By these means, athletes with unusual deviations from predicted performances are identified and made subject for testing using blood testing. Moreover, structured PO analyses with inter-personal scope have been performed to study the possible influence from natural variations in hormone levels on athletic performance. To test associations between serum androgen levels and performance, athletes have been classified in tertiles according to their free testosterone (fT) concentration and the best competition results achieved in the highest and lowest fT tertiles then compared [33]. When contrasted with the lowest female fT tertile, women with the highest natural fT tertile performed significantly better in 400 m, 400 m hurdles, 800 m, hammer throw, and pole vault, with margins of 2–4%. The results have been used by the International Association of Athletics Federation (IAAF) to conclude that female athletes with high fT levels have a significant competitive advantage over those with low fT in the corresponding events. A question for future research is whether there is an opportunity to even more accurately represent performance in circumstances such as the two mentioned above using a contextualised result analysis.

### 4.2. Expanding the Scope of PO Evaluations

The performance metric categories of ‘rank’ and ‘crude measurement’ identified in the review have been shown to display limitations when used as singular endpoints for evaluation. Evaluating crude measurements has been challenged in its narrow approach to the evaluation of ‘success’, particularly with respect to ‘losing’ and ‘failure’ [34]. Correspondingly, the calibre of competition at an event may result in overvalued or undervalued rankings. An athlete may continually lose narrowly to the highest-calibre athletes and be poorly ranked; conversely, an athlete may continually beat competitors of lower calibre and be ranked unduly high [21]. Applying a contextual framework to a performance metric enables the evaluation of the resulting PO to move from a singular endpoint with limitations to a more versatile endpoint that captures and considers a broader array of factors. This process is important to coalesce the varied constructs that result in the PO, yet the challenge remains in quantifying the PO beyond identifying and describing the constructs that inform it.

An alternative approach to evaluation of POs is to base these on subjective goals. Subjective evaluation of performance metrics or contextual results as endpoints alone has limitations. Subjective seasonal goals could take many forms, including a ‘mastery’ approach whereby an athlete is motivated by the achievement of absolute or intra-personal competence or avoidance of incompetence, or conversely, a ‘performance’ approach whereby the athlete is motivated to do better than or not do worse than others [35]. Performance approach goals may lack stability over time, as ‘goal switching’ is thought to be more prevalent in goals that are established under reasons of ‘external pressure’ compared with ‘autonomous reasons’ [36]. Subjective goals evaluated by this approach may be challenged through questioning the veracity or appropriateness of the goal itself. A competition of higher standard may then shift the subjective evaluation of individual performance from a ‘crude measurement’ like finishing time to the ‘rank’ of finishing place [37]. Athletes may also avoid self-definitions of failure by obtaining satisfaction or success through smaller accomplishments [38]. Linking the evaluation of POs to the attainment of a subjective goal therefore has questionable value in the objective categorical evaluation of performance.

### 4.3. Practice Implications

Effective PO evaluation in sports practice settings, for instance, by a national federation, may require that a contextual result be analysed using a combination of constructs. Examples of constructs derived from combining contextual results include: Deviations from a trend of intra-personal crude measurements like height or distance, surpassing a performance standard like intra-personal season’s best, or reference to an inter-personal meet ranking or national record. Each construct described in these examples may be analysed as a singular endpoint; however, a comprehensive evaluation of PO would consider the contribution of each of these endpoints in combination. The concept chart provides a methodology to objectify PO, yet further work is required to develop a ‘next step’ in the objective concept chart methodology to encapsulate a combination of constructs like the examples described above for effective evaluation.

### 4.4. Review Strengths and Limitations

Strengths in this scoping review include the use of a highly structured method consistent with a systematic review process yet flexible where required within the scoping review. This allowed the identification and description of the various methods used in athletics to describe PO as an endpoint for evaluation. We delimited our search criteria to benchmark events in athletics, and more research may exist when assessing a broader array of events. We were thus able to clearly identify limitations in the current body of work towards objectifying PO and gaps in the literature that open opportunity to further research.

Having considered several alternative review methodologies, we found that the scoping review methods incorporating systematic processes were the current best practice to identify and report on research using POs as endpoints. An unexpected limitation was the lack of existing research that satisfied the triage criteria for quantitative data extraction. We searched a common array of academic research databases; however, the inclusion of a greater number of databases and also comprising ‘grey literature’ may have enhanced the possibility of finding further research on the topic. The thematic analysis was conducted systematically and according to well-established methods. This iterative analysis was qualitative by nature and generalisability in a strict meaning cannot thus be regarded as one of its expected attributes. A pragmatic approach to validating qualitative analyses was instead adopted: Use of systematic sampling, triangulation and constant comparison, and proper audit and documentation [39].

## 5. Conclusions

The motivation to undertake this work was to address a ‘real-world’ desire to minimise possible subjectivity in performance evaluation processes. Through a scoping review and thematic analysis, we have described the existing foundation for an objective methodology towards categorising and analysing performance metrics from IBE’s in athletics. By objectifying and establishing PO as an ultimate endpoint for sports medicine and epidemiological research, further opportunities evolve to apply this methodology to sports federation athlete evaluation and the investigation of factors that influence PO success and failure. A considerable challenge remains in constructing a methodology that combines the two observed independent performance metric variables (continuous and ordinal) that are currently utilised as endpoints.

## Figures and Tables

**Table 1:** Characteristics of studies included for qualitative analysis plus thematic analysis and sports practice labels.

Authors	Study Design	Country	Study Population	Study Aims	CATEGORY PERFORMANCE METRIC (Continuous/Ordinal)	THEME FRAMEWORK (Intra-/Inter-personal scope)	CONSTRUCT ANALYSIS (Deviation/Association)	Risk of Bias	
Gernigon and Delloye (2003) [20]	RCT	France	62 National level sprinters (42 male and 20 female). Age 19.9 ± 3.1 years	“…to examine the influence of an unexpected success or failure on a first sprint trial on elite sprinters’ self-efficacy and performance on a second trial immediately following.”	**Continuous: IAAF points table score** 60 m trial raw result converted to points via IAAF points scoring table [29]	INTRA-Personal	**Deviation—from Intra-personal trend** Comparison between two 60 m trials	Downs and Black	17/31 (55%)
Auersperger et al. (2009) [22]	Prospective cohort	Slovenia	47 Middle distance runners 6 senior, 14 junior, 14 youth, 12 boys	“…to show an expert model for the prediction of middle-distance runners’ competitive success and at the same time to establish the relationship between the given potential model of success (assessment of expert modelling) and the athlete’s competitive performance (criterion variable).”	**Ordinal: Personal best** Average of personal best in each of 800 m, 1000 m, and 1500 m converted to IAAF points [29] = ‘criterion variable’. Criterion variable correlated with 17 independent variables within each athlete.	INTRA-Personal	**Association**—correlation between the criterion variable (average of IAAF points in each event) and 17 independent variables.	NOS	5/9
Beggs et al. (2017) [21]	Prospective cohort	UK	33 male sprinters competing in the Diamond League 2015	“We therefore designed the study presented here, with the specific aim of using the PageRank algorithm to evaluate the relative performance of male 100 m sprinters throughout the course of the 2015 IAAF Diamond League season.”	**Ordinal: Finishing position** Finishing position per athlete contesting the 100 m A race at each Diamond League event.	INTER-personal	**Deviation—from inter-personal performance standard** Comparison of algorithmic race result rankings compared with Diamond league points ranking.	NOS	5/9
Chapman et al. (2014) [25]	Prospective cohort	USA	121 Elite track and field athletes (55 male, 66 female)	“…to determine if functional movement screen scores were related to season’s-best performance changes over a 2-year period in elite track and field athletes.” and “To make comparisons between sexes and different events…”	**Ordinal: Season’s best** SB in primary event from 2 consecutive seasons. **Ordinal: Ranking with respect to (wrt) National record** For event and sex comparison SB was normalised to a % of the American record.	INTRA-Personal INTER-personal	**Association**—correlation between % change of SB performance and the functional movement screen score. **Deviation—from inter-personal performance standard** Comparison between groups by sex and event	NOS	4/9
Stoeber and Crombie (2010) [19]	Prospective cohort	UK	161 (103 male, 58 female) University athletes. Age 20.7 ± 2.3 years	“…to investigate whether the contrast between performance approach and performance avoidance goals would predict competitive performance in sports other than triathlon.”	**Continuous: IAAF points table score** Raw result from the first event converted to IAAF points [30] **Ordinal: Finishing position** ‘Qualification success’: Yes/no regarding qualification to the next round or final	INTRA-Personal INTER-personal	**Association**—correlation between performance outcome (IAAF points) and achievement goal approach **Association**—correlation of qualification success with achievement goal approach	NOS	4/9
Thomas et al. (1983) [28]	Prospective cohort	USA	44 male collegiate athletes (24 distance runners, 20 sprinters and jumpers) Age 17–22	“In the present study, selected physiological and psychological factors were examined in order to determine their relationship to track and field performance.”	**Ordinal: Ranking wrt World record** SB result (1980) as a % of the world record (WR)	INTRA-Personal	**Association**—correlation between SB as % of WR and independent physiological and psychological variables	NOS	5/9
Bilic and Smajlovic (2012) [18]	Retrospective Case Report	Bosnia and Herzegovina	1 Heptathlete	“…to offer an efficient model and tools to the athletic practice that allow to achieve an objective, scientific and methodologically based model for an individual analysis and determination of the typical structure of heptathlon disciplines of the particular heptathlete and its structures of the interrelationships among the athletic heptathlon disciplines, as a factor of importance for the development and maximal performance of their own potential.”	**Continuous: IAAF points table score** Heptathlon points scoring table	INTRA-Personal	**Deviation—from intra-personal trend** Career performance trajectory at major championships		
Boccia et al. (2017) [23]	Retrospective Case Series	Italy	200 annual best long and high jump athletes over 10-year period aged 12 to 35	“…to examine the career trajectories of Italian high and long jumpers to provide a better understanding of performance development in jumping events.”	**Ordinal: Season’s and Personal best** Annual SB and career PB over a 10-year period.	INTRA-Personal	**Deviation—from intra-personal trend** Annual rate of change of SB and age of achieving PB		
Haake et al. (2014) [24]	Retrospective descriptive correlational	UK	Male and Female Top 25 annual running event performances from 1890 to 2012	“…to use appropriate data and analysis techniques to quantify the relative size of influences on performance in running”.	**Ordinal: Season’s best** SB from annual top 25 performers	INTRA-Personal	**Deviation—from inter-personal trend** Annual rate of change of average of top 25 SB’s **Association**—correlation between the trajectory of the mean top 25 SB’s with reported influences on performance from seven independent variables		
Haake et al. (2015) [27]	Retrospective descriptive correlational	UK	Male and Female Top 25 annual field event performances from 1890 to 2012	“…to use appropriate data and analysis techniques to quantify the relative size of influences on performance in field events”.	**Ordinal: Season’s best** Season’s best (SB) from annual top 25 performers	INTRA-Personal	**Deviation—from inter-personal trend** Annual rate of change of average of top 25 SB’s **Association**—correlation between the trajectory of the mean top 25 SB’s with reported influences on performance from seven independent variables		
Coquart et al. (2009) [26]	Diagnostic	France	330 adult male distance athletes over 5 years. Age 21–50	“…to compare predicted performance by the nomogram from the performance at 2 other distances with actual performance at distances ranging from 10 km to the marathon.”	**Ordinal: Season’s best** Season’s best (SB) from athletes that completed 3 events in the same year (10 km, 20 km and marathon)	INTRA-Personal	**Deviation—from intra-personal performance standard** Extrapolated or interpolated prediction of one distance from the other two performances		

Risk of bias tools: Downs and Black checklist; a score of ≥75% was deemed to be of high quality, 60–75% moderate quality, and ≤60% low quality [15]; NOS = Newcastle–Ottawa scale, a nine-star rating system divided into three categories (Appendix B) [17].

## Appendix A

Literature search strategies

Sports Discus

(((DE “athletics” or TX “Track and Field” or SD “Olympic*” OR SD “World Championships”) and (SU “Athletic Performance” OR SD “Achievement” OR SU “Program Evaluation” or SU “Performance evaluation” or SD “success factors” or TX “SUCCESS” or SU “ACHIEVEMENT” or SD “Program Evaluation” or SD “Performance evaluation” or SD “scoring” or SD “ranking”))) NOT CHILD*

PubMed

(athletics[Title/Abstract] OR “track and field”[Text Word] OR “Olympics”[All Fields] OR “world championships”[All Fields]) AND (“athletic performance”[MeSH Terms] OR “achievement”[MeSH Terms] OR “program evaluation”[MeSH Terms] OR “performance evaluation”[All Fields] OR “success factors”[All Fields] OR success[Text Word] OR scoring[Text Word] OR ranking[Text Word] NOT “child”[All Fields])

## Appendix B

**Table A1:** Risk of bias assessment results.

Article	Score %	Interpretation
Gernigon and Delloye (2003)	55% (17/31)	High risk of bias

**Table A2:** Risk of bias tool Downs and Black checklist; a score of ≥75% was deemed to be of high quality, 60–75% moderate quality, and ≤60% low quality [15].

Articles	Selection/4	Comparability/2	Outcome/3
Auersperger et al., 2009	★★		★★★
Beggs et al., 2017	★★		★★★
Chapman et al., 2014	★		★★★
Stoeber and Crombie 2010	★		★★★
Thomas et al., 1983	★★		★★★

NOS = Newcastle–Ottawa scale, a nine-star rating system divided in to three categories [16].

## References

[1] Van Yperen N.W., Leander N.P. (2014). The overpowering effect of social comparison information: On the misalignment between mastery-based goals and self-evaluation criteria. Personal. Soc. Psychol. Bull..

[2] Caglar E., Canlan Y., Demir M. (2009). Recreational exercise motives of adolescents and young adults. J. Hum. Kinet..

[3] Hodge K., Allen J.B., Smellie L. (2008). Motivation in Masters sport: Achievement and social goals. Psychol. Sport Exerc..

[4] Van Mechelen W., Hlobil H., Kemper H.C. (1992). Incidence, severity, aetiology and prevention of sports injuries. Sports Med..

[5] Brooks J.H., Fuller C.W. (2006). The influence of methodological issues on the results and conclusions from epidemiological studies of sports injuries. Sports Med..

[6] Raysmith B.P., Drew M.K. (2016). Performance success or failure is influenced by weeks lost to injury and illness in elite Australian track and field athletes: A 5-year prospective study. J. Sci. Med. Sport.

[7] Drew M.K., Raysmith B.P., Charlton P.C. (2017). Injuries impair the chance of successful performance by sportspeople: A systematic review. Br. J. Sports Med..

[8] Engebretsen L., Soligard T., Steffen K., Alonso J.M., Aubry M., Budgett R., Dvorak J., Jegathesan M., Meeuwisse W.H., Mountjoy M. (2013). Sports injuries and illnesses during the London Summer Olympic Games 2012. Br. J. Sports Med..

[9] Gratton C., Rowe N., Veal A. (2011). International comparisons of sports participation in European countries: An update of the COMPASS project. Eur. J. Sport Soc..

[10] Tricco A.C., Lillie E., Zarin W., O’Brien K.K., Colquhoun H., Levac D., Moher D., Peters M.D., Horsley T., Weeks L. (2018). PRISMA Extension for Scoping Reviews (PRISMA-ScR): Checklist and Explanation. Ann. Intern. Med..

[11] Shamseer L., Moher D., Clarke M., Ghersi D., Liberati A., Petticrew M., Shekelle P., Stewart L.A. (2015). Preferred reporting items for systematic review and meta-analysis protocols (PRISMA-P) 2015: Elaboration and explanation. BMJ.

[12] Arksey H., O’Malley L. (2005). Scoping studies: towards a methodological framework. Int. J. Soc. Res. Methodol..

[13] Levac D., Colquhoun H., O’Brien K.K. (2010). Scoping studies: advancing the methodology. Implement. Sci..

[14] Braun V., Clarke V. (2006). Using thematic analysis in psychology. Qual. Res. Psychol..

[15] Downs S.H., Black N. (1998). The feasibility of creating a checklist for the assessment of the methodological quality both of randomised and non-randomised studies of health care interventions. J. Epidemiol. Community Health.

[16] Wells G., Shea B., O’Connell D., Peterson J., Welch V., Losos M., Tugwell P. (2009). The Newcastle-Ottawa Scale (NOS) for Assessing the Quality of Nonrandomised Studies in Meta-Analyses.

[17] Munn J., Sullivan S.J., Schneiders A.G. (2010). Evidence of sensorimotor deficits in functional ankle instability: A systematic review with meta-analysis. J. Sci. Med. Sport.

[18] Bilić M., Smajlović N. (2012). Model for longitudinal analysis of an individual all-rounder athlete’s potential. Homo Sport.

[19] Stoeber J., Crombie R. (2010). Achievement goals and championship performance: Predicting absolute performance and qualification success. Psychol. Sport Exerc..

[20] Gernigon C., Delloye J.-B. (2003). Self-Efficacy, Causal Attribution, and Track Athletic Performance Following Unexpected Success or Failure Among Elite Sprinters. Sport Psychol..

[21] Beggs C.B., Shepherd S.J., Emmonds S., Jones B. (2017). A novel application of PageRank and user preference algorithms for assessing the relative performance of track athletes in competition. PLoS ONE.

[22] Auersperger I., Ulaga M., Škof B. (2009). An expert model for determining success in middle-distance running./Ekspertni model uspešnosti za teke na srednje proge. Kinesiol. Slov..

[23] Boccia G., Moise P., Franceschi A., Trova F., Panero D., La Torre A., Rainoldi A., Schena F., Cardinale M. (2017). Career Performance Trajectories in Track and Field Jumping Events from Youth to Senior Success: The Importance of Learning and Development. PLoS ONE.

[24] Haake S.J., Foster L.I., James D.M. (2014). An improvement index to quantify the evolution of performance in running. J. Sports Sci..

[25] Chapman R.F., Laymon A.S., Arnold T. (2014). Functional Movement Scores and Longitudinal Performance Outcomes in Elite Track and Field Athletes. Int. J. Sports Physiol. Perform..

[26] Coquart J.B., Alberty M., Bosquet L. (2009). Validity of a nomogram to predict long distance running performance. J. Strength Cond. Res..

[27] Haake S., James D., Foster L. (2015). An improvement index to quantify the evolution of performance in field events. J. Sports Sci..

[28] Thomas T.R., Zebas C.J., Bahrke M.S., Araujo J., Etheridge G.L. (1983). Physiological and psychological correlates of success in track and field athletes. Br. J. Sports Med..

[29] Spiriev B., Spiriev A. (1998). IAAF Scoring Tables of Athletics, Revised Edition.

[30] Spiriev B., Spiriev A. (2008). IAAF Scoring Tables of Athletics, Revised Edition.

[31] Hopker J., Schumacher Y.O., Fedoruk M., Mørkeberg J., Bermon S., Iljukov S., Aikin R., Sottas P.-E. (2018). Athlete performance monitoring in anti-doping. Front. Physiol..

[32] Malcata R.M., Hopkins W.G. (2014). Variability of competitive performance of elite athletes: A systematic review. Sports Med..

[33] Bermon S., Garnier P.-Y. (2017). Serum androgen levels and their relation to performance in track and field: mass spectrometry results from 2127 observations in male and female elite athletes. Br. J. Sports Med..

[34] Dyck N. (1995). Parents, consociates and the social construction of children’s athletics. Anthropol. Forum.

[35] Elliot A.J., McGregor H.A. (2001). A 2 × 2 achievement goal framework. J. Personal. Soc. Psychol..

[36] Vansteenkiste M., Lens W., Elliot A.J., Soenens B., Mouratidis A. (2014). Moving the achievement goal approach one step forward: Toward a systematic examination of the autonomous and controlled reasons underlying achievement goals. Educ. Psychol..

[37] Thiel C., Foster C., Banzer W., De Koning J. (2012). Pacing in Olympic track races: Competitive tactics versus best performance strategy. J. Sports Sci..

[38] Podlog L. (2002). Perceptions of Success and Failure Among University Athletes in Canada. J. Sport Behav..

[39] Finfgeld-Connett D. (2010). Generalizability and transferability of meta-synthesis research findings. J. Adv. Nurs..

